# Supplementary material for: Vitamin D Modified DSS-Induced Colitis in Mice via STING Signaling Pathway
Source: Biology (Basel). 2025 Jun 18;14(6):715. doi: 10.3390/biology14060715 (PMC12190092; doi:10.3390/biology14060715)
Supplement: Supplementary file 1 [file biology-14-00715-s001.zip › Supplementary Materials S1: 16s-report of gut microbiota/Groups/diff_analysis/RF/METACYC_Groups.RFimportance.pdf]

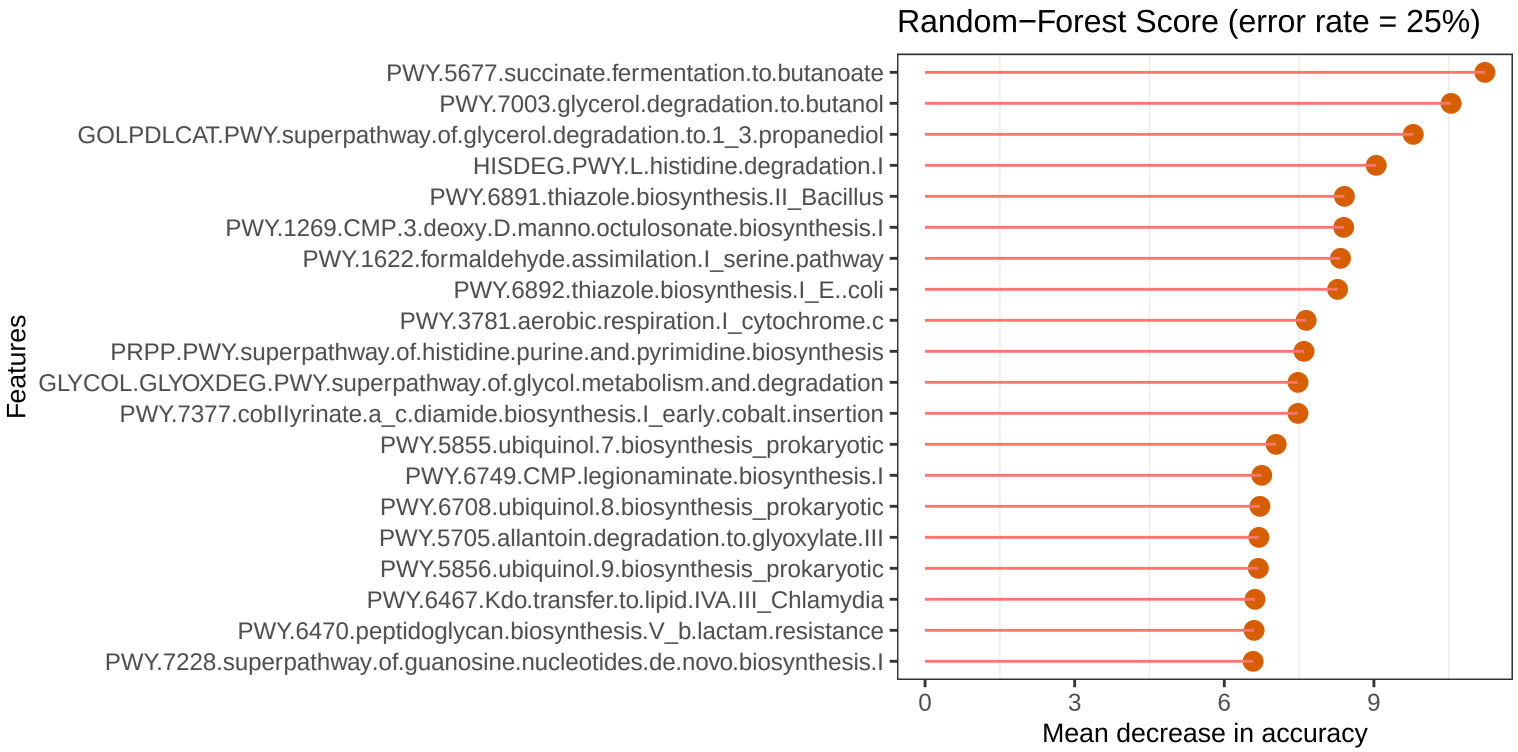
Random−Forest Score (error rate = 25%)
Features
PWY.5677.succinate.fermentation.to.butanoate
PWY.7003.glycerol.degradation.to.butanol
GOLPDLCAT.PWY.superpathway.of.glycerol.degradation.to.1_3.propanediol
HISDEG.PWY.L.histidine.degradation.I
PWY.6891.thiazole.biosynthesis.II_Bacillus
PWY.1269.CMP.3.deoxy.D.manno.octulosonate.biosynthesis.I
PWY.1622.formaldehyde.assimilation.I_serine.pathway
PWY.6892.thiazole.biosynthesis.I_E..coli
PWY.3781.aerobic.respiration.I_cytochrome.c
PRPP.PWY.superpathway.of.histidine.purine.and.pyrimidine.biosynthesis
GLYCOL.GLYOXDEG.PWY.superpathway.of.glycol.metabolism.and.degradation
PWY.7377.cobIlyrinate.a_c.diamide.biosynthesis.I_early.cobalt.insertion
PWY.5855.ubiquinol.7.biosynthesis_prokaryotic
PWY.6749.CMP.legionaminate.biosynthesis.I
PWY.6708.ubiquinol.8.biosynthesis_prokaryotic
PWY.5705.allantoin.degradation.to.glyoxylate.III
PWY.5856.ubiquinol.9.biosynthesis_prokaryotic
PWY.6467.Kdo.transfer.to.lipid.IVA.III_Chlamydia
PWY.6470.peptidoglycan.biosynthesis.V_b.lactam.resistance
PWY.7228.superpathway.of.guanosine.nucleotides.de.novo.biosynthesis.I
0
3
6
9
Mean decrease in accuracy
